# A Word to the Wise Is Sufficient

**Published:** 1897-08

**Authors:** 


### A Word to the Wise is Sufficient.

The *Medical Record*, of January 23, 1897, is responsible for the following: A pleasing custom exists in a town in Devonshire, England. At Christmas-time all the medical men in the place invite those who have been under their professional care during the year, those who are still living, at least, to dine with them. After all have eaten at the doctor's expense, the cigars and hot toddy are set out, and then comes the turn of the hosts. Each one corrals his own flock and proceeds to administer a corrective in the shape of a gentle reminder of the indebtedness of each head of the family. The victims are prepared, however, and settle up without demur, and the new year begins properly.

